# Wisconsin State Medical Society

**Published:** 1850-09

**Authors:** Geo. D. Wilber

**Affiliations:** Rec. Sec’y.


### ARTICLE XI.

### THE WISCONSIN STATE MEDICAL SOCIETY

Met in Milwaukee on the 19th inst.,.and convened in the City Hotel to hold its annual session.

Dr. H. Van Dusen, from the Western Medical Society of the State of Wisconsin, and Drs. T. K. Bartlett, G. Wright, T. B. Selby, and —. Warner, from the Milwaukee County Medical Society, were admitted to the membership of the Society as delegates.

On motion, an invitation was extended to the regular physicians and surgeons now in the city to take seats in the Society, and participate in its deliberations during the present session.

On motion, the Secretaries were instructed to prepare and issue letter circulars to the regular practitioners of medicine and surgery throughout the State, requesting them to be present in Madison on the third Wednesday of January, A. D., 1850, to attend a Mass Medical Convention, there and then to be holden, which has for its object the establishment of the profession of medicine upon a respectable footing, by securing the passage of a law by the Legislature for that purpose, and the transaction of such other business as may be thought expedient.

Dr. Geo. D. Wilber and Dr. J. Johnson were, on motion, elected permanent members of the Society.

The annual election of officers resulted as follows :—

Dr. ALFRED L. CASTLEMAN, of Delafield, President.
" H. VAN DUSEN, Mineral Point, Vice-President.
" Geo. D. Wilber, Mineral Point, Recording Secretary.
" James P. Whitney, Milwaukee, Corresponding Sec'y.
" T. B. Dousman, Milwaukee, Treasurer.

Dr. G. Wright, Waukesha,
" T. K. Bartlett, Milwaukee, } *Censors.*
" J. Johnson, Milwaukee,

On motion, the President was requested to deliver an address before the Society at the close of his term of office, on the subject of Medical Reform.

On motion, Resolved, That the next annual meeting of this Society be held in Madison, on the second Tuesday of June, A. D., 1851.

Ordered by the Society, that an abstract of the proceedings of this meeting be furnished to the public prints in the State for publication.

Adjourned to meet in Madison on the third Wednesday of January, A. D., 1851, at 12 o'clock, M.

GEO. D. WILBER, *Rec. Sec'y.*

